# CRISPR/Cas9 or prime editing? – It depends on…

**DOI:** 10.1515/biol-2020-0109

**Published:** 2020-12-03

**Authors:** Dirk Schenke

**Affiliations:** Department of Molecular Phytopathology and Biotechnology, Christian-Albrechts-University Kiel, Hermann-Rodewald Straße 9, 24118 Kiel, Germany

**Keywords:** crop resistance breeding, CRISPR/Cas, prime editing

Genome editing (GE) emerges to become an indispensable tool in basic research and crop breeding. This owes especially to clustered regularly interspaced short palindromic repeats (CRISPR)/Cas, the newest kind of site-specific nucleases derived from an ancient bacterial immune system against foreign DNA [1]. New improvements are published almost weekly, and basically, CRISPR/Cas-based GE technologies can be distinguished into two categories in terms of introducing either random or distinct mutations at the target site: the first category comprises CRISPR/Cas9 (but also Cas12 or other CRISPR type 1 systems) causing random mutations at the target site when double strand breaks (DSBs) are repaired by non-homologous end joining (NHEJ). The second category consists of precise genome editing technologies such as prime editing [2], base editors [3] and CRISPR/Cas systems applied in combination with donor templates to repair DSBs by the technically more challenging homology directed repair (HDR) pathway [1]. Thus, researchers and breeders can soon rely on “educated guess” while deciding which change to introduce within their gene of interest (GOI). At this point, there are several choices: change can mean to correct a non-functional allele restoring a common phenotype or mimic natural variation detected in other species. Functional genomics comparing genomes and transcriptomes of different organisms allows the identification of suitable natural polymorphisms in genes relevant to plant–pathogen interactions. Successful applications are for example polymorphisms detected in the eIF4E gene from pea, which were mimicked in Arabidopsis to create virus resistance [4] or in promoters such as the rice bsr-d1 promoter where a single base change enhanced binding of MYBS1 to downregulate a peroxidase gene, thereby increasing H_2_O_2_ production and Blast resistance [5]. There is indeed a bright future for GE applications in the breeding stress resilient crops, especially in light of climate change which increases both abiotic and biotic stress situations. However, biotic stress might be easier to address because during co-evolution between host and pathogen very specific mechanisms were established rendering the host resistant or allowing the pathogen to overcome this resistance. The latter mechanisms deployed by pathogens largely involve so-called effectors, which can be proteins, RNAs or DNA having only one purpose which is to manipulate the host [6,7]. The high specificity underlying effector–target recognition allows deploying GE to prevent pathogen-caused resistance breaking. One example is the meanwhile well-established strategy to knock-out (KO) crop susceptibility factors, thereby increasing resistance [8]. These host factors are required by the pathogen to successfully colonize its host and upon their loss susceptibility will be reduced. However, GE is currently predominantly deployed in a way that causes random mutations in the target gene, and thus there can be deletions or insertions multiple of 3 bps, which cause no frame-shift mutation and thereby probably no visible change in the phenotype. With HDR or prime editing, it should be possible to induce specifically 1 or 2 bp insertions or deletions to guarantee a KO phenotype. This would be a clever way to prevent that several of the GE-induced mutations are without clear phenotype. But the KO of susceptibility factors is generally not free of side effects given that host genes do not just exist for manipulation by the pathogens, but have a certain physiological function during plant development. However, due to the degenerated nature of the genetic code it is also possible to induce tailored changes, which will not negatively affect crop physiology and cause a trade-off.

One example is to mutate the promoter cis-elements targeted, e.g. by bacterial transcription activator-like effectors (TALEs) to induce the expression of susceptibility genes [9] or to rewrite target sequences of small non-coding RNAs (sRNAs) deployed by pathogens during cross-kingdom RNAi in order to harness the hosts silencing machinery for degradation of its own resistance genes [8,10]. For such purposes, prime editing appears currently as the method of choice since it allows specific induction of insertions (currently up to 15 nt) and deletions (up to 40 nt) as shown for rice and wheat [11] or multiple base mutations as achieved also in rice [12]. Moreover, small changes within a 17 bp limit are indistinguishable from naturally occurring sequences in any larger genome (a random sequence of that size would be found statistically once in 4^17^ nucleotides, which equals about 17 Gb – the genome size of wheat), and considering that among 80 *Arabidopsis thaliana* accessions from various geographic regions >800,000 naturally occurring small InDels (up to 20 nt) were identified [13], it can be postulated that also InDel mutations of this size are quite natural. Importantly, such small GE-induced mutations should be more than sufficient to disrupt TALE binding (which requires ca. 18 bp recognition sites; [14]), or cross-kingdom RNAi relying on sRNAs with an average size of 22 nt [10,15]. These are therefore good examples how GE can be deployed to increase genetic variation for resistance breeding without any trade-offs to be expected (Figure 1).

**Figure 1:**
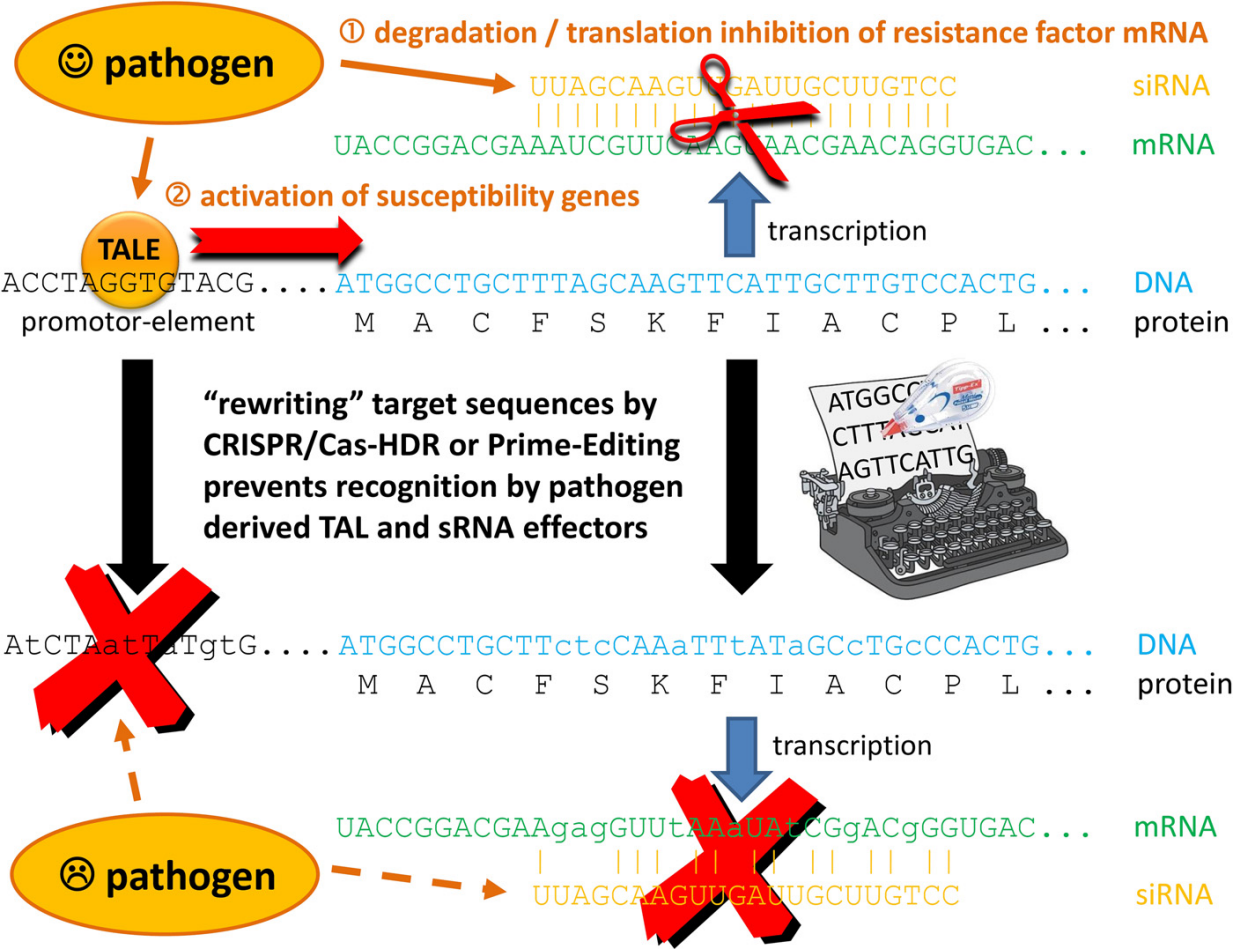
Rewriting of just 17 bp sequences with prime editing can have a big impact on the outcome of the plant–pathogen interaction. (1) Pathogen-derived siRNAs that target host resistance genes during cross-kingdom RNAi can be disarmed by rewriting their target nucleotide sequence without affecting the encoded amino acid sequence due to the degenerated nature of the genetic code. (2) Promoter elements can be changed, so that in the case of susceptibility genes these are not recognized by bacterial TALEs anymore, while establishing such a cis-sequence for example in the promoter of an immunity gene should trigger a defense reaction in the presence of the TALE-producing pathogen (not indicated).

## Give chance a chance

Genetic variation depends in nature on spontaneous random mutations as the driving force of evolution via selection and researchers as well as plant breeders should not completely abandon methods involving random mutagenesis. Introducing precisely defined mutations by prime editing is surely an advantage in the cases discussed above, but if CRISPR/Cas is applied in reverse genetics to modify a GOI, e.g. a susceptibility gene to increase resistance to a pathogen, it might be advisable to do it by classical CRISPR/Cas9. With this method the target gene is mutated randomly, albeit at a desired position. One example is the discovery of a new *CRT1a* allele combination in *Brassica napus* (oilseed rape), which was found in search of novel susceptibility factors to render this crop resistant to the fungal pathogen *Verticillium longisporum* [16]. Since *B. napus* is amphidiploid, this gene exists in four copies, which slightly vary in their amino acid sequence depending on their origin within the AA genome (originated from *Brassica rapa*) or the CC genome (from *Brassica oleracea*). One CRISPR/Cas9-induced mutant showed best performance when the AA-genome copies were mutated, whereas the CC-genome copies were left unchanged. Of course the construct was designed to mutate all four alleles, but by chance this combination showed the best resistance phenotype. Furthermore, both AA-genome alleles displayed different mutations, one causing a frameshift, the other one a 3 bp deletion leading to loss of Phe8. This unique situation would have never been created by “educated guess”. Therefore, it is not necessarily wrong to give chance a chance when the intention is to increase natural variation for breeding purposes. The question is only: do you feel lucky?
